# Draft Genome Sequence of the Entomopathogenic Bacterium *Bacillus pumilus* 15.1, a Strain Highly Toxic to the Mediterranean Fruit Fly *Ceratitis capitata*

**DOI:** 10.1128/genomeA.01019-15

**Published:** 2015-09-24

**Authors:** Diana C. García-Ramón, Leopoldo Palma, Colin Berry, Antonio Osuna, Susana Vílchez

**Affiliations:** a Institute of Biotechnology, Campus Fuentenueva, University of Granada, Granada, Spain; b Facultad de Ciencias Agrarias, Universidad Nacional del Litoral, Esperanza, Santa Fe, Argentina; c Consejo Nacional de Investigaciones Científicas y Técnicas, Buenos Aires, Argentina; d Cardiff School of Biosciences, Cardiff University, Cardiff, Wales, United Kingdom; e Department of Biochemistry and Molecular Biology I, Campus Fuentenueva, University of Granada, Granada, Spain

## Abstract

We present the draft whole-genome sequence of the entomopathogenic *Bacillus pumilus* 15.1 strain that consists of 3,795,691 bp and 3,776 predicted protein-coding genes. This genome sequence provides the basis for understanding the potential mechanism behind the toxicity and virulence of *B. pumilus* 15.1 against the Mediterranean fruit fly.

## GENOME ANNOUNCEMENT

*Bacillus pumilus* strain 15.1 has been recently described to be toxic against larvae of the Mediterranean fruit fly, *Ceratitis capitata*, one of the most damaging pests for fruits and vegetables worldwide (1). Strain 15.1 was isolated from a partially decomposed reed plant and has recently been characterized microbiologically and biochemically (D. C. Garcia-Ramon, C. A. Molina, A. Osuna, S. Vilchez, submitted for publication). *B. pumilus* strain 15.1 was deposited in the Spanish Type Culture Collection as CECT 7462.

*B. pumilus* strains are known to have very interesting properties, such as being highly resistant to environmental stresses (2) and producing a wide range of metabolites of industrial value. Nevertheless, only a few genomes from this species have been published. We report the first genome sequence of an entomopathogenic *B. pumilus* strain.

DNA from *B. pumilus* 15.1 was sequenced on a HiSeq 2000 sequencing system (Illumina Sequencing) in a single-read mode with a read length of 50 bases (GATC Biotech, Constance, Germany). The whole-genome sequencing yielded 26,322,535 reads. The reads were assembled in contigs by using Velvet software (3) with the *de novo* assembly tool and default parameters and then by iterative mapping using Geneious Pro R8 software, obtaining 63 contigs. The genome size of *B. pumilus* was 3,795,691, with a G+C content of 41.3%. Genome annotation was added by the NCBI Prokaryotic Genome Annotation Pipeline (PGAP), although it was also analyzed with BLAST (4) using a custom insecticidal toxin database (5, 6). A total of 3,776 protein-coding genes, 45 pseudogenes, 2 rRNA genes, 16 tRNA genes, and 1 noncoding RNA gene were predicted in the *B. pumilus* 15.1 genome.

*B. pumilus* 15.1 bears at least two extrachromosomal elements, one plasmid and one megaplasmid (D. Garcia-Ramon, M. J. Luque-Navas, C. A. Molina, C. del Val, A. Osuna, and S. Vilchez, submitted for publication). The plasmid sequence (7,785 bp, 35.7% G+C content) corresponds to contig 38 in the assembly. The megaplasmid has not yet been assigned to any contig or contig sequences, but it has been detected in total DNA extractions on agarose gels (D. Garcia-Ramon et al., submitted). The *B. pumilus* 15.1 genome also contains prophage elements, even though a genome analysis revealed the presence of a clustered regularly interspaced short palindromic repeat (CRISPR)/Cas system (bacterial innate immune mechanism for protection from foreign DNA) (7). Interestingly, during sporulation this strain forms parasporal crystals that morphologically resemble those produced by *Bacillus thuringiensis* Cry proteins (D. C. Garcia-Ramon, C. A. Molina, A. Osuna, S. Vilchez, submitted for publication). The role of these crystalline structures has not yet been elucidated, and we have detected no *cry*-like gene coding sequences in the genome. However, the *B. pumilus* 15.1 genome harbors other genes encoding well-known entomopathogenic factors, such as chitinases (8), metalloproteases (9), and cytolysins (10).

The analysis and characterization of the *B. pumilus* 15.1 genome will provide new insights to allow the elucidation of the pathogenic mechanism of *B. pumilus* 15.1 against the Mediterranean fruit fly, providing useful information for the development of novel biotechnological products suitable for the control of this pest and the discovery of unknown virulence factors of this entomopathogenic bacterium.

### Nucleotide sequence accession numbers.

This whole-genome shotgun project has been deposited at DDBJ/EMBL/GenBank under the accession number LBDK00000000. The version described in this paper is version LBDK00000000.1.
